# The Functional Role of Loops and Flanking Sequences of G-Quadruplex Aptamer to the Hemagglutinin of Influenza a Virus

**DOI:** 10.3390/ijms22052409

**Published:** 2021-02-27

**Authors:** Anastasia A. Bizyaeva, Dmitry A. Bunin, Valeria L. Moiseenko, Alexandra S. Gambaryan, Sonja Balk, Vadim N. Tashlitsky, Alexander M. Arutyunyan, Alexey M. Kopylov, Elena G. Zavyalova

**Affiliations:** 1 Chemistry Department, Lomonosov Moscow State University, 119991 Moscow, Russia; bunin_dm@mail.ru (D.A.B.); valerian.moiseenko@gmail.com (V.L.M.); tashlitsky@belozersky.msu.ru (V.N.T.); kopylov.alex@gmail.com (A.M.K.); 2 Chumakov Federal Scientific Centre for Research and Development of Immune and Biological Products RAS, 108819 Moscow, Russia; al.gambaryan@gmail.com; 3 Forte’Bio, Fremont, CA 94538, USA; sonja.balk@moldev.com; 4 Belozersky Research Institute of Physical Chemical Biology, Lomonosov Moscow State University, 119991 Moscow, Russia; alarut@genebee.msu.ru

**Keywords:** DNA aptamer, G-quadruplex, influenza virus, hemagglutinin, affinity, structure−activity relationship

## Abstract

Nucleic acid aptamers are generally accepted as promising elements for the specific and high-affinity binding of various biomolecules. It has been shown for a number of aptamers that the complexes with several related proteins may possess a similar affinity. An outstanding example is the G-quadruplex DNA aptamer RHA0385, which binds to the hemagglutinins of various influenza A virus strains. These hemagglutinins have homologous tertiary structures but moderate-to-low amino acid sequence identities. Here, the experiment was inverted, targeting the same protein using a set of related, parallel G-quadruplexes. The 5′- and 3′-flanking sequences of RHA0385 were truncated to yield parallel G-quadruplex with three propeller loops that were 7, 1, and 1 nucleotides in length. Next, a set of minimal, parallel G-quadruplexes with three single-nucleotide loops was tested. These G-quadruplexes were characterized both structurally and functionally. All parallel G-quadruplexes had affinities for both recombinant hemagglutinin and influenza virions. In summary, the parallel G-quadruplex represents a minimal core structure with functional activity that binds influenza A hemagglutinin. The flanking sequences and loops represent additional features that can be used to modulate the affinity. Thus, the RHA0385–hemagglutinin complex serves as an excellent example of the hypothesis of a core structure that is decorated with additional recognizing elements capable of improving the binding properties of the aptamer.

## 1. Introduction

Nucleic acid aptamers are known as molecular recognizing elements, with high specificity and affinity. The functional activity of aptamers is mediated by the specific structures of the oligonucleotides. The search for aptamer structure–activity relationships (SARs) remains a focus of fundamental interest. Several aptamer–protein complexes have been identified [1,2,3,4,5,6]. Here, one of the more interesting examples is described, revealing the core structure of the aptamer that binds influenza hemagglutinin (HA).

HA is the major glycoprotein found on the influenza viral particle surface [7] and mediates the attachment and penetration of the virus into the host cells. Therefore, HA is an attractive target for the development of antiviral agents and recognizing elements. A number of DNA and RNA aptamers have been identified that bind HA from various influenza strains [8], including DNA aptamers with G-tracts [9,10,11], which are able to form G-quadruplexes. The G-quadruplex is a noncanonical nucleic acid structure, which consists of stacked guanine tetrads stabilized by cation chelation and hydrogen bonds between guanine bases [12]. Previously, the G-quadruplex structure was found in some aptamers to different protein and small molecule targets [13,14,15,16]. The G-quadruplex aptamer RHA0385 (5′-TTG GGG TTA TTT TGG GAG GGC GGG GGT T-3′), was selected against the HA of the A/Anhui/1/05 (H5N1) influenza virus strain [9]. More than that, RHA0385 has been shown to recognize other influenza A viral strains in spite of low sequence homology [17]. This attribute was used to create RHA0385-based aptasensors for influenza A viruses [17,18,19]. The tertiary structure of the aptamer might bind with some protein sites that are conserved among the different strains; another possibility is that the geometrical shape of the G-quadruplex matches the HA surfaces and this suitability acts as the primary driving force of complex formation.

**Figure 1:**
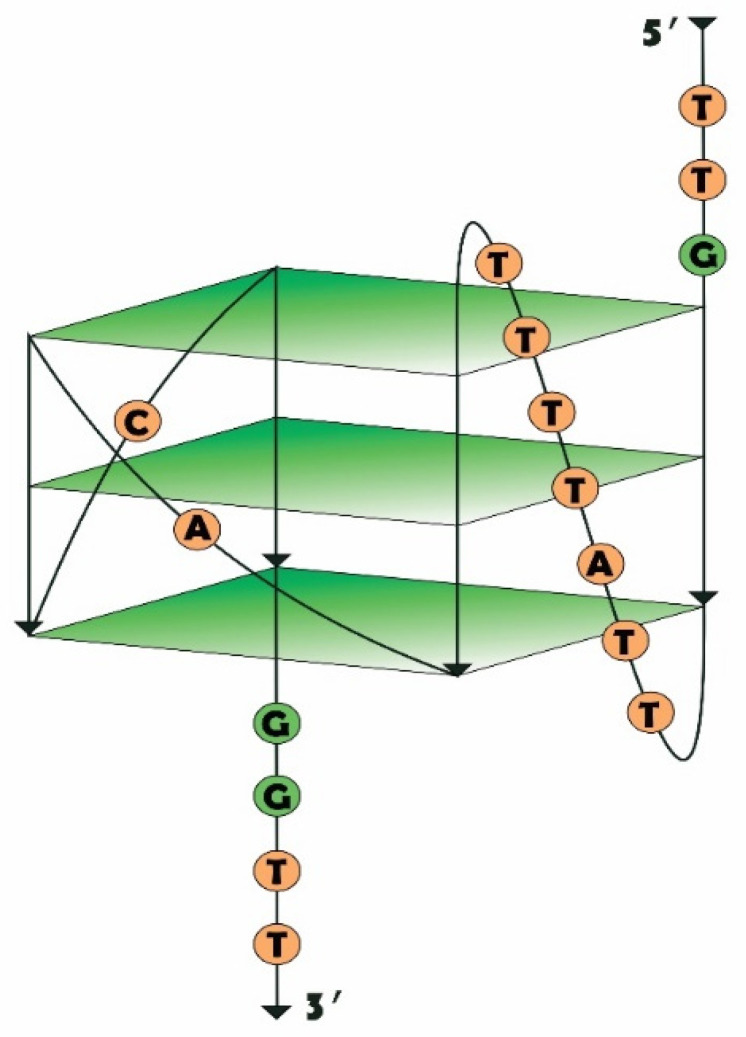
The proposed structure of RHA0385 as a parallel G-quadruplex with three propeller loops; one of them is 7 nucleotides long (a major loop), whereas two others are single-nucleotide (minor loops).

The proposed structure of RHA0385 is a parallel G-quadruplex composed of three G-tetrads featuring three propeller loops; one loop is 7 nucleotides long (a major loop), whereas the other two are each a single nucleotide in length (minor loops). The G-quadruplex has short 5′- and 3′-flanking sequences (Figure 1) [20]. Three derivatives of RHA0385 featuring alterations in the major loop were generated and examined in our previous study. Alterations of this loop affected the stability of the G-quadruplex and its conformational homogeneity, although the high affinity and broad specificity against various influenza A strains were retained [20]. In this study, we broadened the dataset of RHA0385 variants to investigate the role of all loops and flanking sequences on the RHA0385 affinity to the HA. We also aimed to identify the minimal core structure of the aptamer necessary to retain HA affinity. Recombinant HA (rHA) and influenza A viruses (i.e., hemagglutinin on the influenza viral particle surface, vHAs) of the H5N1 strains were chosen for the affinity experiments. The structure and affinity of a set of minimal G-quadruplexes were characterized, revealing a minimal core structure that is able to recognize HA.

## 2. Results and Discussion

### 2.1. Design of RHA0385 Variants

To establish the roles played by different parts of the aptamer in its structure and function, three sets of RHA0385 variants (Table 1) were constructed. The proposed earlier structure of RHA0385 as a G-quadruplex, with three G-tetrads and three 7:1:1-nucleotide propeller loops, and the 5′- and 3′-flanking sequences (Figure 1, [20]) comprised the initial structure of these series of RHA0385 variants.

The first set of variants was constructed to test the necessity of the flanking sequences. Aptamers G7n3t and G7n5t lack the 3′-end (GGTT) and 5′-end (TTG) of RHA0385, respectively, whereas aptamer G7nt has no flanking sequences on either end. The proposed flanking sequences for RHA0385 contain a G at the 5′-end and a GG at the 3′-end, so the exact guanines which participate in the RHA0385 structure currently remain unknown, although this study might provide some clues to address this question.

The second set was designed to determine whether the first 7-nucleotide loop of RHA0385 can be truncated down to one nucleotide. These variants were designed using the G7nt as an initial sequence, which lacked any flanking sequences and contained only single-nucleotide loops; therefore, the possibility of a minimally functional, 15-nucleotide, G-quadruplex able to bind HA was also explored. G7nt and RHA0385 already contain two single-nucleotide loops (Figure 1); thus, only the major loop was shortened. The nucleotides T, A, and C were considered for these loops because G can participate in the G-quadruplex structure. The names given to these derivatives were as follows: G7nt-X_Y_Z, where the major loop (loop 1) is represented by nucleotide X, loop 2 is represented by nucleotide Y, and loop 3 is represented by nucleotide Z.

The third set of RHA0385 variants contained recently studied structures [20], including mutations and deletions from the major loop, with the flanking sequences retained. G7-TTATTAA_A_C contains more purines in the major loop than the initial RHA0385 aptamer (major loop sequence: TTATTTT), and the major loop of the G7-TTA_A_C aptamer was truncated up to the first 3 nucleotides.

### 2.2. Affinity of RHA0385 and Its Variants to Viral and Recombinant HA

Biolayer interferometry (BLI) is a biosensor technique intended to monitor complex association and dissociation under real-time conditions and provide data for the calculation of kinetic and equilibrium constants [21]. BLI has been successfully applied to study aptamer-protein complexes [22,23].

**Figure 2:**
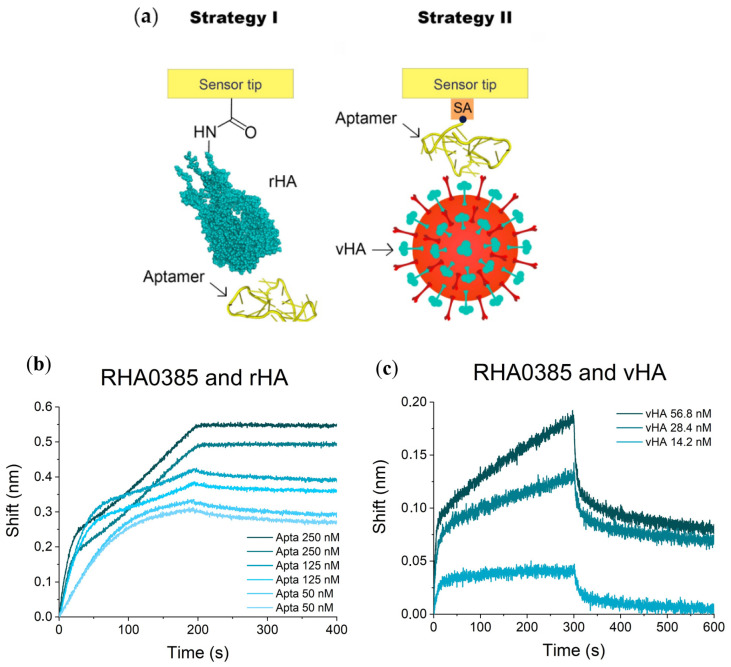
BLI binding assays of aptamer to HA of influenza A virus: (**a**) two strategies of the experiment with immobilization of recombinant HA and immobilization of the aptamer. Influenza virus picture was taken from Kisscc0 website [24]; sensorgrams for the complex of RHA0385 aptamer with (**b**) rHA and (**c**) vHA.

The two different strategies were implemented to study the formation of aptamer–HA complexes (Figure 2a). Strategy I involved the immobilization of rHA on amine-reactive sensors. The sensors were then dipped into various concentrations of aptamer solutions to obtain sensorgrams. Strategy II used streptavidin-coated sensors with aptamers immobilized via a biotin moiety on the 5′-end of the oligonucleotide, with the influenza virus in the solution.

The sensorgrams for the complexes of the RHA0385 aptamer and rHA from the H5N1 virus are shown in Figure 2b. The inflections at the association curve are the result of a complex mechanism of aptamer binding that is distinct from the Langmuir 1:1 model of interaction. We used the first part of the association curve to perform further calculations. The dissociation constant, aK_D_, for the complex between RHA0385 and rHA was 0.6 ± 0.3 nM (Table 2), which was significantly lower than previously published constants measured by surface plasmon resonance (SPR; 13 ± 3 nM [20]). This discrepancy is due to the kinetic dissociation constant, which was 45 times slower in this experiment compared with previous data. The most probable explanation is that some surface effects were induced by the use of different polymers used during chip functionalization procedures by the manufacturers. A similar apparent increase in the affinity was observed between thrombin and a thrombin binding aptamer in BLI experiments compared with other measures [6,25].

The sensorgrams for complexes of the RHA0385 aptamer and the vHA of the H5N1 virus are shown in Figure 2c. The dissociation constant, aK_D_, was 0.05 ± 0.02 nM (Table 2), which was also significantly lower than the value from a previously published enzyme-linked aptamer assay (aK_D_ = 8 ± 3 nM [20]). The observed 100-fold reduction in the constant could be the result of the multipoint binding of the virus to the aptamer-functionalized surface in the BLI experiment, whereas in the enzyme-linked aptamer assay, the virions were immobilized and applied to aptamer solutions.

**Figure 3:**
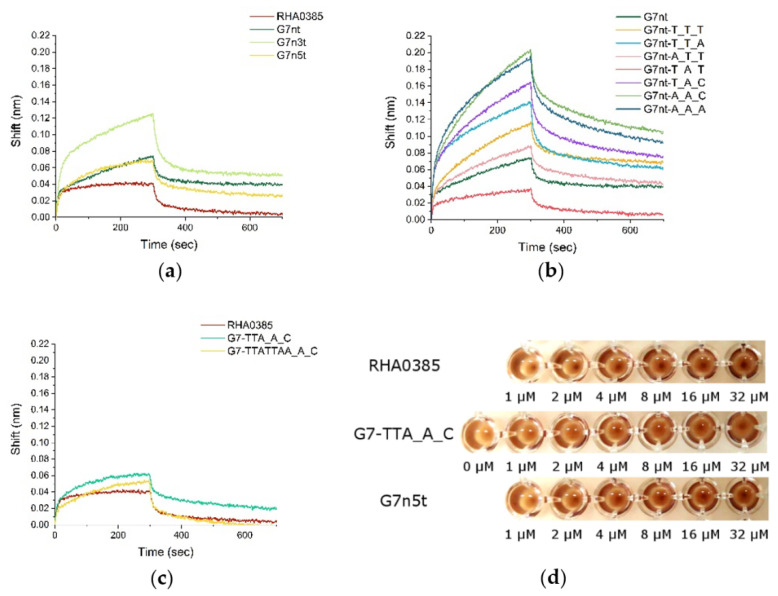
Aptamer binding vHA measured by BLI (concentration of vHA was set as to 14 nM): (**a**) RHA0385 and aptamers with flanking sequences truncated; (**b**) G7nt and aptamers with single-nucleotide loops; (**c**) RHA0385 and aptamers with major loop altered. (**d**) Hemagglutination inhibition assay of 3.6 nM vHA with RHA0385, G7-TTA_A_C and G7n5t at different concentrations.

The three sets of oligonucleotides were tested using both strategies (Figure 3, Supplementary Figures S1 and S2, Table 2). Surprisingly, all of the oligonucleotides studied had affinities to both rHA and vHA. The aK_D_ values for rHA ranged from 0.3 to 0.8 nM, suggesting that the structural changes had only minor effects on the ability to bind to the recombinant protein. The highest affinity was achieved for G7n5t, whereas the lowest affinity was observed for G7nt-T_T_A. In these two examples, affinity was enhanced and diminished primarily due to changes in the kinetic association constants, which were significantly lower for the single-nucleotide major loop structures.

When examining the affinity to viral particles, the differences in aK_D_ values were much more pronounced. The lowest aK_D_ values were observed for G7n5t and G7-TTA_A_C (0.04 nM for each). The worst affinity was observed for G7nt-T_T_A (0.7 ± 0.3 nM). All variants with the single-nucleotide major loop, except for G7nt-T_A_T, presented with considerably reduced affinity. The decrease in the kinetic association constant represented the primary contribution to the decreased affinity. These results clearly indicated that the major loop contributes to the recognition of the functionally active protein as a part of a viral particle, whereas the parallel G-quadruplex represents a core structure with a basic level of affinity for HA.

The inactivation of vHA by aptamer was demonstrated by hemagglutination inhibition (HAI) assay. The RHA0385 inhibited hemagglutination even at 1 µM concentration (Figure 3d), while for G7n5t and G7-TTA_A_C, the same effect was observed at higher concentrations (2 µM and 4 µM, respectively). The aptamers with the same affinities to vHA possessed the different efficacy in HAI, and these preliminary data showed the significance of the major loop and flanking sequence in the inhibition of influenza HA binding to the host cell.

### 2.3. G-Quadruplex Structure of Aptamers

**Figure 4:**
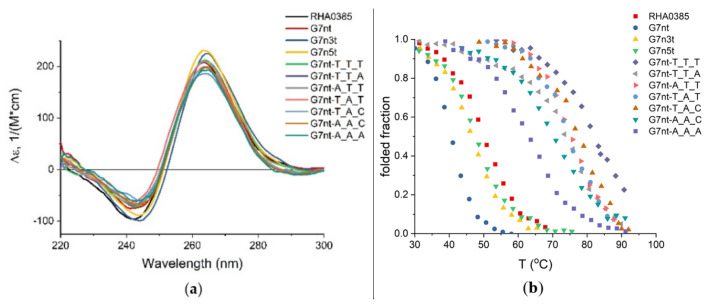
Circular dichroism spectroscopy of aptamers to HA: (**a**) CD spectra of RHA0385 and its variants obtained at the same conditions (20 °C, 2 µM solution in sodium-potassium phosphate buffer pH 7.4 in the presence of 10 mM K^+^); (**b**) normalized curves of the CD melting of RHA0385 and its variants.

We attempted to identify the reasons for the differences in the affinity associated with the structural peculiarities of the various G-quadruplexes. Firstly, the set of oligonucleotides was analyzed using circular dichroism (CD) spectroscopy. The CD spectra with a positive maximum near 264 nm and a negative maximum at 245 nm are characteristic of a parallel G-quadruplex [26,27,28,29]; this type of structure was observed for RHA0385 and all of its derivatives (Figure 4a).

The melting temperatures (T_m_) of the G-quadruplexes were calculated from CD melting curves (Figure 4b, Table 3). After the melting process, annealing was performed to check the presence of hysteresis. Strictly speaking, no hysteresis was found under experimental conditions, but the conformations before and after the melting–annealing process can be clearly discerned by different intensities of the CD maximum (see example in Supplementary Figure S3). This observation, as well as conformational heterogeneity (see Section 2.4), did not allow the calculation of the thermodynamic parameters, thus only the thermal stability of the aptamers can be compared. All of the examined G-quadruplexes had T_m_ values over 40 °C, suggesting that they were stable at room temperature. The truncation of the flanking sequences decreased the melting temperature, which agreed with previous data [30].

G-quadruplexes with single-nucleotide loops are very stable, and the T_m_ values for these structures were higher than 60 °C. Single-nucleotide loops are known to be associated with high T_m_ values [31,32]. For example, for G7nt-T_T_T, the T_m_ was not able to be calculated exactly due to the rather high temperatures, therefore the T_m_ value was estimated to be higher than 80 °C. Previous experimental research using similar conditions has reported a T_m_ as high as 92 °C [33].

### 2.4. Oligomeric Composition of G-Quadruplexes

Size-exclusion high-performance liquid chromatography (SE-HPLC) is a convenient tool for the distinction of intra- and intermolecular G-quadruplex structures [34,35,36]. The oligomeric compositions of the G-quadruplexes were estimated by SE-HPLC, as described by Alieva et al. [37,38]. Previously, RHA0385, G7-TTATTAA_A_C, and G7-TTA_A_C were shown to be predominantly intramolecular G-quadruplexes under the conditions used for the affinity experiments [20] (Table 3).

The normalized SE-HPLC chromatograms for the RHA0385 derivatives are shown in Supplementary Figure S5. The ratios between the experimental molecular weight (M_exp_) and the theoretical molecular weight (M) are shown. The M_exp_ value was calculated using the calibration curve (Supplementary Figure S4). The peaks for M_exp_/M ranging from 0.8 to 1.3 are thought to correspond with intramolecular G-quadruplexes. The contents of the intramolecular G-quadruplexes are listed in Table 3.

The G-quadruplexes with a long major loop (3 or 7 nucleotides) were predominantly intramolecular (more than 74% of monomeric species). The G-quadruplexes with single-nucleotide major loops had large contents of a tetrameric species (31%–57%), along with intramolecular species. This observation agreed with previous data regarding the intermolecular structures of three-quartet G-quadruplexes with short loops [32]. The binding constants of G-quadruplexes with single-nucleotide major loops could be affected by the presence of intermolecular structures.

### 2.5. Correlations between Structure and Affinity of RHA0385 Derivatives

**Figure 5:**
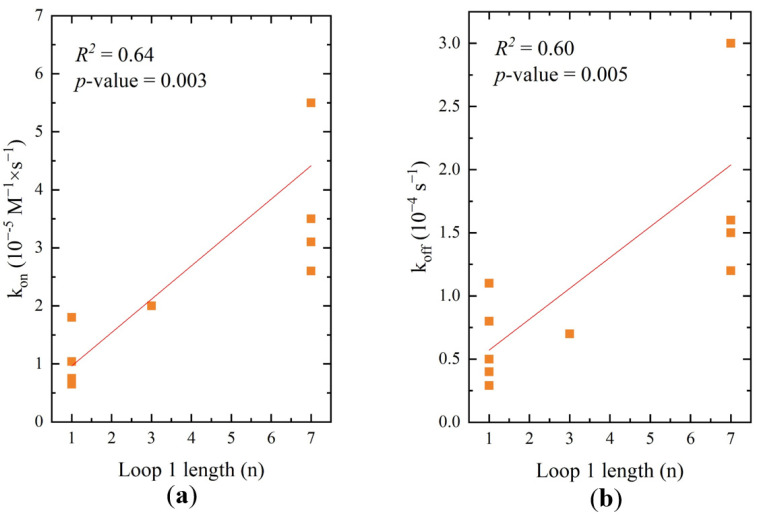
The plots of rate constants (**a**) association rate constants, (**b**) dissociation rate constants versus the aptamer’s loop length for complexes aptamer–rHA.

Two structural parameters were chosen for correlation analysis. The first parameter was the length of the major loop, and the second parameter was the content of intramolecular G-quadruplexes. The equilibrium and kinetic constants were chosen as the affinity parameters. The equilibrium constants were represented in logarithmic form, as Gibbs free energy of binding, ΔG_b,303_. The dependencies were approximated with linear regression. The coefficients of determination (*R*^2^) are given in Table 4, as are the *p*-values for F-statistics. This analysis was performed for both affinity experiments, but only the aptamer–rHA complexes yielded relatively strong correlations, with *R*^2^ values higher than 0.6 [39] and *p*-values less than or equal to 0.005 [40] (Figure 5 and Table 4).

The length of the major loop affected both kinetic constants. Faster association (Figure 5a) and faster dissociation (Figure 5b) were observed for aptamers with large major loops, including the original aptamer, RHA0385. Due to contradictory trends in the kinetic constants, correlations between structural parameters and ΔG_b,303_ values were not found. No significant correlations between the monomer contents and affinity parameters were found.

Based on these results, as well as on the previous discussion here, HA was likely recognized by the parallel G-quadruplex topology itself. The flanking sequences and loops appeared to tune the affinity of the aptamers. For example, a large major loop (≥3 nucleotides) increased the kinetic association constant, accelerating complex formation. The selectivity of HA toward different G-quadruplexes was increased for the viral particles compared with the recombinant proteins, which could be due to differences in the HA state [41]. The data for viral particles is more relevant for further consideration, as aptamers are commonly used as recognition elements for whole viral particles [42].

It should be noted that the G7nt-T_T_T aptamer variant is just the same as the interleukin (IL)-6 receptor aptamer AID-1-T (GGGTGGGTGGGTGGG [43]), the HIV-1 integrase aptamer T30922 (GGGTGGGTGGGTGGG [44]); moreover, it is similar to a set of oligonucleotides with antiproliferative activity [45]. Therefore, one more target protein for this G-quadruplex structure was found. This oligonucleotide cannot be considered to be an aptamer with high target specificity. Instead, the G7nt-T_T_T represents a core structure that can be modified to become a specific aptamer against a concrete protein. The flanking sequences, loop alterations, and modular construction could be used in the optimization of each aptamer and provide specificity for a single target protein.

## 3. Materials and Methods

### 3.1. Oligonucleotides Preparation

All oligonucleotides samples were in sodium-potassium phosphate buffer pH 7.4 (8 mM Na_2_HPO_4_, 137 mM NaCl, 1.5 mM KH_2_PO_4_, 8.5 mM KCl, all salts are from Helicon, Russia), prepared in fresh deionized MilliQ water (obtained with MilliPore equipment from Merck, Darmstadt, Germany), then filtered through a membrane with 0.22 μm pores (MilliPore, Darmstadt, Germany), and degassed. Unmodified and 5′-biotinylated RHA0385 and its derivatives, as well as control oligonucleotide (CG ACG CAC CAT TTG TTT AAT ATG TTT TTT AAT TCC CCT TGT GGT GCG TCG, 50 nt.), and the oligonucleotides for SE-HPLC column calibration (GGT TGG TGT GGT TGG—15 nt., 4712 kDa; GGG TTT GGG TTG GGT TGG G—19 nt., 6435 kDa, GGT TGG TGT GGT TGG TGG TTG GTG TGG TTG G—31 nt, 9788 kDa; TTG GGG TTA TTT TGG GAG GGC GGG GGT TTT TTT TGG GGT TAT TTT GGG AGG GCG GGG GTT—60 nt., 18,887 kDa) were synthesized and purified by Evrogen LLC (Moscow, Russia), then stored at −20 °C in 1 mM stock solution in deionized MilliQ water (obtained with MilliPore equipment from Merck, Darmstadt, Germany). Immediately before each experiment, the oligonucleotide was diluted to 2 μM in a phosphate buffer pH 7.4. For G-quadruplex formation, each sample was heated to 95 °C, held for 5 min and cooled to room temperature.

### 3.2. Influenza A Virus Preparation and Characterization

Influenza A viruses of A/chicken/Kurgan/3654at/2005 (H5N1) strain, and chicken and hamster red blood cells were provided by the Chumakov Federal Scientific Centre for Research and Development of Immune and Biological Products of the Russian Academy of Sciences. The viruses were grown in eggs and inactivated by the addition of 0.05% (*v/v*) glutaraldehyde (Helicon, Moscow, Russia), and 0.03% (*w/w*) NaN_3_ (Helicon, Moscow, Russia) was added as a preservative. Immediately before each experiment the virus sample was purified from insoluble impurities by centrifugation at 2000 rpm, +4 °C for 5 min (centrifuge Eppendorf 5417R, Hamburg, Germany) and then transferred into phosphate buffer pH 7.4 for a threefold repeat of centrifugation at 16,400 rpm for 10 min and subsequent dissolving of precipitate in the same volume of phosphate buffer pH 7.4.

The standard protocol of hemagglutination test was used (VIRAPUR, San Diego, CA, USA). V-bottom 96-well plates were from Greiner Bio-One, Kremsmünster, Austria. In each well 50 μL phosphate buffer pH 7.4 was added. In the first column, 50 μL of virus sample was added and then was serially diluted by half across 11 wells of a 96-well plate. A portion of 50 μL of 0.5% chicken red blood cells was then added to each well and incubated at room temperature for 1 h. The hemagglutination titer was read as the last well in the serial dilution that did not form a red button of settled RBCs. Virus loads in viral particle per mL (VP/mL) were estimated from hemagglutination units (HAU/mL) based on correlations published previously [46]. The vHA trimers concentration was calculated with the assumption that there were approximately 300 HA trimers on a virion [7].

### 3.3. BLI Affinity Assay

The binding affinities of RHA0385 and its derivatives were determined by biolayer interferometry (BLI) assay on a ForteBIO Octet Red96 instrument (ForteBIO, Menlo Park, CA, USA) and Data Acquisition software v.10.0 (ForteBio, CA, USA) at 30 °C. Samples were placed in a 96-well black plate (Greiner Bio-One, Kremsmünster, Austria) in a 300 μL/well volume, then agitated at 1000 rpm during the experiment. The two strategies were developed to study the affinities of aptamers to rHA and vHA (Figure 2a).

#### 3.3.1. I Strategy (rHA Immobilized + Aptamer in Solution)

Carboxyl groups of biosensors intended for the amine coupling reaction (AR2G type, ForteBio, Menlo Park, CA, USA) were activated for 5 min in the solution of 100 mM EDAC (1-ethyl-3-(3ʹ-dimethylaminopropyl)carbodiimide, Roth, Germany) and 100 mM s-NHS (N-Hydroxysulfosuccinimide sodium salt, Chem-Impex Int’l, Wood Dale, IL, USA), then loaded in a 5 μg/mL recombinant HA1 subunit of H5 from A/Vietnam/1203/2004 (H5N1) strain (ab190125, Abcam, Burlingame, CA, USA) in 40 mM phosphate buffer pH 6.4 for 10 min. Five out of eight tips within a row were loaded with protein while three were immersed into phosphate buffer pH 7.4 only and were used as control for baseline drifting. The remaining carboxyl groups were deactivated with the 1 M ethanolamine hydrochloride (Sigma-Aldrich, Darmstadt, Germany) pH 8.5 for 5 min. After the signal stabilization in the phosphate buffer pH 7.4, the association stage was performed. After preformation, the aptamer 2 μM solution was diluted consequently into 1000, 500, 250, 125, or 50 nM solutions in phosphate buffer pH 7.4. The five loaded sensors were immersed into these aptamer solutions and the remaining three sensors were dipped into the phosphate buffer pH 7.4 only. All sensors were placed in the phosphate buffer pH 7.4 during the dissociation stage. Both association and dissociation were monitored for 200 s each. The regeneration of the sensors was conducted by the threefold repeat of immersing sensors into 1 M ethanolamine hydrochloride pH 8.5, for 15 s and the phosphate buffer pH 7.4, for 15 s. The reference signal was obtained at the next round of the experiment. The same loaded sensors were immersed into the 50 nM solution of control DNA oligonucleotide (CG ACG CAC CAT TTG TTT AAT ATG TTT TTT AAT TCC CCT TGT GGT GCG TCG), which does not bind HA. Each experiment was performed twice. Signals from loaded sensors were baseline corrected, then the signal from the reference was subtracted.

#### 3.3.2. II Strategy (vHA in Solution + Aptamer Immobilized)

All aptamers were chemically biotinylated at the 5′-end allowing immobilization onto an SA coated sensor surface (ForteBIO, Menlo Park, CA, USA). Each aptamer was annealed as described earlier, then diluted to 100 nM in a phosphate buffer pH 7.4. Four out of eight tips within a row were saturated with 100 nM biotinylated aptamers for 400 s, and four tips were loaded with phosphate buffer pH 7.4 only. Viral particles of A/chicken/Kurgan/3654at/2005 (H5N1) strain were prepared in the phosphate buffer pH 7.4 as a dilution series in duplicate (typically in the range from 14 to 114 nM per vHA trimer) along with the buffer blanks. Association was monitored for 300 s and dissociation was followed for 600 s in the phosphate buffer pH 7.4 alone. The regeneration of the sensors was conducted by the threefold repeat of immersing sensors into 1 M ethanolamine hydrochloride pH 8.5, for 15 s and the phosphate buffer pH 7.4, for 15 s. The reference signal was obtained from the sensors loaded with control DNA oligonucleotide 5′-biotin-CG ACG CAC CAT TTG TTT AAT ATG TTT TTT AAT TCC CCT TGT GGT GCG TCG, which does not bind HA. Signals from loaded sensors were baseline corrected, then the signal from reference was subtracted. Each experiment was performed twice.

The sensorgram processing was performed manually with the OriginPro 2020 software in agreement with the Langmuir 1:1 binding model [47]. Values of the rate constants of complex association (k_on_) and dissociation (k_off_) were determined using the exponential approximations of the sensorgrams. Dissociation rate constants were calculated from fitting the experimental dissociation curves (from 2 to 200 s) with exponential function Exp2PMod1, OriginPro 2020 software (S = a × exp(bt), where S is a shift (nm), a is a constant, −b is a dissociation rate constant k_off_, t is a time (s)). Association phase (the first 60 s for aptamer–rHA complexes and the first 20 s for aptamer–vHA complexes) was fitted with the BoxLucas1 exponential function, OriginPro software (S = a_1_ × (1 − exp(−b_1_t)), where S is a shift (nm), a_1_ is a constant, b_1_ = k_on_C + k_off_, t is a time (s)). The parameters b_1_ of each sensorgram were plotted against concentration of the counterpart in solution, C (M), and the outlying dots were removed. The linearisation of this plot for each aptamer–protein complex yielded an association rate constant k_on_ as the slope. The goodness of the fit was judged by the *R*^2^ values approaching 1 (see Supplementary Figure S6). Apparent dissociation constants aK_D_ were calculated from the equation aK_D_ = k_off_/k_on_. Gibbs free energy of complex formation was calculated by equation = −RT × ln(aK_D_), where T = 303 K for the affinity experiments.

### 3.4. HAI Test

The influenza A viruses of A/chicken/Kurgan/3654at/2005 (H5N1) strain (8 HAU, or 3.6 nM vHA) in phosphate buffer pH 7.4 was mixed with aptamers diluted in the range 1–32 µM (in total 100 µL vHA–aptamer solution per well of the V-bottom 96-well plates (Greiner Bio-One, Kremsmünster, Austria)). The 100 µL of 0.5% hamster red blood cells were added to each well and incubated at room temperature for 1 h.

### 3.5. Circular Dichroism (CD) Spectroscopic Study

CD spectra of oligonucleotides were obtained with a Chirascan spectrometer (Applied Photophysics Ltd., Leatherhead, Surrey, UK) equipped with a temperature controller. The spectrum of phosphate buffer pH 7.4 at 20 °C was used for a baseline correction. The oligonucleotides were tested at 2 µM strand concentration. After the above-described preformation the aptamer sample was transferred into a quartz cell of 1 mm optical path length for CD measurement. The initial spectra were recorded at 20 °C and at wavelengths of 220–300 nm. The melting process was performed by heating the aptamer sample from 20 °C to 92.5 °C at a rate of 0.5 °C/min with the spectra registration at each 2.5 min. Immediately after melting, the annealing process was performed for each oligonucleotide by cooling from 92.5 °C to 20 °C at the same rate. Each experiment was performed twice on the different samples of each aptamer. Each circular dichroism ΔA value was baseline corrected and divided by the oligonucleotide concentration to generate molar circular dichroism ∆ε. To obtain melting and annealing curves, the positive maximum of the CD spectra (264 nm) was followed. The normalized melting curves were obtained by plotting of folded fraction vs. T (°C). The folded fraction of the G-quadruplex was calculated for each temperature T by the equation:

folded fraction = (Δε_T_−Δε_f_)/(Δε_d_−Δε_f_),

where Δε_T_ is the molar CD at 264 nm at temperature T, Δε_f_ is the molar CD at 263 nm of the folded structure (initial plateau), and Δε_d_ is the molar CD at 263 nm of the denatured structure (final plateau). Aiming to obtain melting temperatures T_m_, the melting curves were approximated with sigmoidal curves of the Boltzmann model with OriginPro 2020 software.

### 3.6. Size-Exclusion High Performance Liquid Chromatography (SE-HPLC)

SE-HPLC was conducted with an Agilent 1200 HPLC system with autosampler and diode array detector (Agilent, Santa Clara, CA, USA) at 25 °C. The HPLC column TSKgel G2000SWXL (Tosoh Bioscience, South San Francisco, CA, USA) was intended for the separation of 5–150 kDa proteins. The column parameters were as follows: 30 cm length, 0.78 cm diameter, 5 µm diameter of particles, 12.5 nm mean pore diameter. The separation was performed at the flow rate of 0.5 mL/min. The mobile phase consisted of water MilliQ and acetonitrile in a 9:1 *v/v* ratio, supplemented with potassium phosphate buffer 11 mM KH_2_PO_4_, 9 mM K_2_HPO_4_, 160 mM Na_2_SO_4_, pH 7.0. Absorption at 260 nm was registered with a 10 nm bandwidth. The calibration of the column and the experiments were performed with set of G-quadruplex oligonucleotides, injected independently (GGT TGG TGT GGT TGG—15 nt., 4712 kDa; GGG TTT GGG TTG GGT TGG G—19 nt., 6435 kDa, GGT TGG TGT GGT TGG TGG TTG GTG TGG TTG G—31 nt, 9788 kDa; TTG GGG TTA TTT TGG GAG GGC GGG GGT TTT TTT TGG GGT TAT TTT GGG AGG GCG GGG GTT—60 nt., 18,887 kDa). The logarithm values from the molecular weights, lgM, were plotted against corrected retention volumes, then linearized. The calibration curve was described by the equation:

lgM = (−2.4 ± 0.2) × V_R_/V_0_ + (7.7 ± 0.3),

with *R*^2^ = 0.99 (Supplementary Figure S4).

## 4. Conclusions

The G-quadruplex aptamer RHA0385 to HA of influenza A virus represents a promising molecular recognition element for use in aptasensors that bind influenza A viruses from different strains. This study sheds light on the structural peculiarities of the aptamer which appear to be responsible for HA recognition. Studying three sets of RHA0385 derivatives revealed that the presence of a parallel G-quadruplex provides the basic affinity for HA and could be considered a minimal core structure. The flanking sequences and alteration of the loops represent additional features that can tune the affinity of the aptamer. These data are of high interest not only for aptamers to HA, but also for other aptamers that feature a similar core structure, such as the aptamers to IL-6 receptor and HIV-1 integrase.

## Figures and Tables

**Table 1:** RHA0385 variants: sequences, lengths, extinction coefficients and molecular weights. The sequences under alteration are shown in bold.

ID	Sequence, 5′-3′	Length, nt	ɛ, cm^−1^ × M^−1^	Mw, Da
RHA0385	**TTG**GGG**TTATTTT**GGG**A**GGG**C**GGG**GGTT**	28	268,100	8834
G7nt	GGGTTATTTTGGGAGGGCGGG	21	205,600	6629
G7n3t	**TTG**GGGTTATTTTGGGAGGGCGGG	24	231,300	7567
G7n5t	GGGTTATTTTGGGAGGGCGGG**GGTT**	25	242,400	7896
G7nt-T_T_T	GGG**T**GGG**T**GGG**T**GGG	15	148,700	4801
G7nt-A_T_T	GGG**A**GGG**T**GGG**T**GGG	15	153,200	4810
G7nt-T_A_T	GGG**T**GGG**A**GGG**T**GGG	15	153,200	4810
G7nt-T_T_A	GGG**T**GGG**T**GGG**A**GGG	15	153,200	4810
G7nt-T_A_C	GGG**T**GGG**A**GGG**C**GGG	15	151,100	4795
G7nt-A_A_C	GGG**A**GGG**A**GGG**C**GGG	15	155,600	4804
G7nt-A_A_A	GGG**A**GGG**A**GGG**A**GGG	15	162,200	4828
G7-TTATTAA_A_C	TTGGGG**TTATTAA**GGGAGGGCGGGGGTT	28	277,900	8851
G7-TTA_A_C	TTGGGG**TTA**GGGAGGGCGGGGGTT	24	235,700	7617

**Table 2:** The affinity parameters for RHA0385 and its variants complexed to vHA (A/chicken/Kurgan/3654at/2005 (H5N1)) and rHA (A/Vietnam/1203/2004 (H5N1)). The values of rate constants of association (k_on_, 1 × 10^5^ M^−1^ × s^−1^) and dissociation (k_off_, 1 × 10^−3^ s^−1^), and apparent dissociation constant (aK_D_, nM) are given.

	I Strategy, rHA			II Strategy, vHA		
	k_on_, 1 × 10^5^ M^−1^ × s^−1^	k_off_, 1 × 10^−4^ s^−1^	aK_D_, nM	k_on_, 1 × 10^5^ M^−1^ × s^−1^	k_off_, 1 × 10^−4^ s^−1^	aK_D_, nM
RHA0385	2.6 ± 0.2	1.5 ± 0.7	0.6 ± 0.3	41 ± 6	2.0 ± 0.6	0.05 ± 0.02
G7nt	3.5 ± 0.2	3 ± 1	0.7 ± 0.3	19 ± 5	2.6 ± 0.6	0.13 ± 0.07
G7n3t	7.4 ± 0.6	3 ± 1	0.4 ± 0.2	28 ± 5	4 ± 1	0.16 ± 0.07
G7n5t	5.5 ± 0.2	1.6 ± 0. 8	0.3 ± 0.2	33 ± 6	1.5 ± 0.5	0.04 ± 0.02
G7nt-T_T_T	1.04 ± 0.04	0.8 ± 0.2	0.7 ± 0.2	8.0 ± 0.8	5 ± 2	0.6 ± 0.3
G7nt-A_T_T	0.65 ± 0.08	0.29 ± 0.09	0.4 ± 0.2	21 ± 4	4 ± 1	0.2 ± 0.1
G7nt-T_A_T	1.80 ± 0.05	1.1 ± 0.4	0.6 ± 0.2	37 ± 9	2.6 ± 0.7	0.07 ± 0.04
G7nt-T_T_A	0.65 ± 0.01	0.5 ± 0.1	0.8 ± 0.2	10 ± 2	7 ± 2	0.7 ± 0.3
G7nt-T_A_C	-	-	-	16 ± 4	5 ± 1	0.3 ± 0.1
G7nt-A_A_C	-	-	-	15 ± 3	4 ± 1	0.3 ± 0.1
G7nt-A_A_A	0.75 ± 0.09	0.4 ± 0.2	0.6 ± 0.4	22 ± 3	5 ± 2	0.2 ± 0.1
G7-TTATTAA_A_C	3.10 ± 0.09	1.2 ± 0.6	0.4 ± 0.2	33 ± 6	3 ± 1	0.09 ± 0.05
G7-TTA_A_C	2.0 ± 0.2	0.7 ± 0.4	0.4 ± 0.2	54 ± 7	2.0 ± 0.3	0.04 ± 0.01

**Table 3:** Content of intramolecular G-quadruplexes according to SE-HPLC and melting temperature of G-quadruplexes (T_m_).

ID	Monomer content, %	T_m_, °C
RHA0385	81 [15]	50.1 ± 0.2 [15]
G7nt	74	40.6 ± 0.1
G7n3t	100	46.5 ± 0.1
G7n5t	74	47.8 ± 0.2
G7nt-T_T_T	60	>80
G7nt-A_T_T	69	75.4 ± 0.3
G7nt-T_A_T	43	74.8 ± 0.5
G7nt-T_T_A	66	78 ± 2
G7nt-T_A_C	60	82 ± 2
G7nt-A_A_C	63	70.3 ± 0.5
G7nt-A_A_A	60	62.9 ± 0.2
G7-TTATTAA_A_C	77 [20]	52.9 ± 0.2 [15]
G7-TTA_A_C	86 [20]	61.4 ± 0.1 [15]

**Table 4:** Relationships between structural and affinity parameters of RHA0385 variants in terms of coefficient of determination (R^2^) values and *p*-values for F-statistics of linear regression (given as follows: *R*^2^/*p*-value. These values are given for the dependencies of kinetic constants of association (k_on_) and dissociation (k_off_), or Gibbs energy of binding (ΔG_b,303_) for aptamer complexes with rHA and vHA versus the different structural parameters of aptamers. The strongest correlations with *R*^2^ ≥ 0.6 and *p*-value ≤ 0.005 are highlighted.

	I strategy, rHA			II strategy, vHA		
	k_on_, 1 × 10^5^ M^−1^ × s^−1^	k_off_, 1 × 10^−4^ s^−1^	ΔG_b,303_, kJ/mol	k_on_, 1 × 10^5^ M^−1^ × s^−1^	k_off_, 1 × 10^−4^ s^−1^	ΔG_b,303_, kJ/mol
Major loop length, nt	0.64/0.003	0.60/0.005	0.22/0.2	0.18/0.1	0.39/0.02	0.35/0.03
Monomer content, %	0.45/0.02	0.27/0.1	0.23/0.1	0.15/0.2	0.08/0.3	0.11/0.3

## Data Availability

The data that support the findings of this study are available from the corresponding authors upon reasonable request.

## Supplementary Materials

The following are available online at https://www.mdpi.com/1422-0067/22/5/2409/s1.

## Abbreviations

BLI	Biolayer interferometry
HA	Hemagglutinin
HAI	Hemagglutination inhibition assay
HPLC	High-performance liquid chromatography
rHA	Recombinant hemagglutinin
vHA	Viral hemagglutinin
SA	Streptavidin
SAR	Structure−affinity relationship
SE-HPLC	Size-exclusion high-performance liquid chromatography
SPR	Surface plasmon resonance
