# Supplementary material for: The Functional Role of Loops and Flanking Sequences of G-Quadruplex Aptamer to the Hemagglutinin of Influenza a Virus
Source: Int J Mol Sci. 2021 Feb 27;22(5):2409. doi: 10.3390/ijms22052409 (PMC7957560; doi:10.3390/ijms22052409)
Supplement: Supplementary file 1 [file ijms-22-02409-s001.pdf]

Supplementary Materials

Supplementary Figure S1. BLI sensorgrams for complexes of aptamers with viral hemagglutinin vHA on viral particles of A/chicken/Kurgan/3654at/2005 (H5N1) strain.

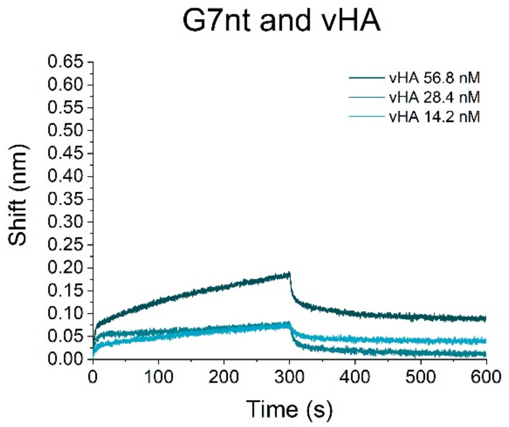

(**a**)

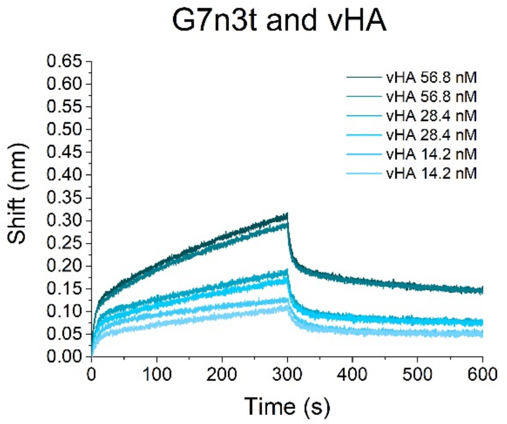

(**b**)

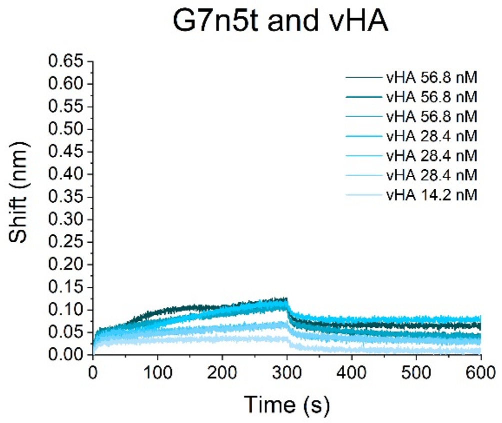

(**c**)

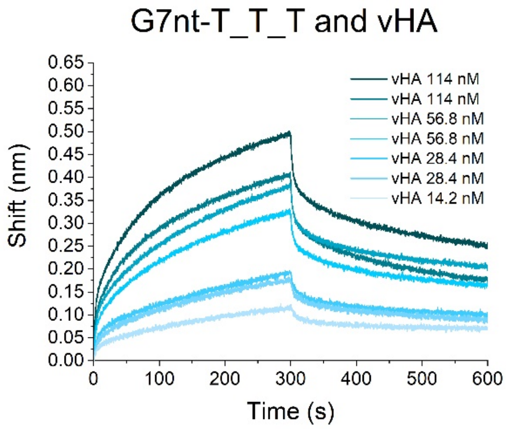

(**d**)

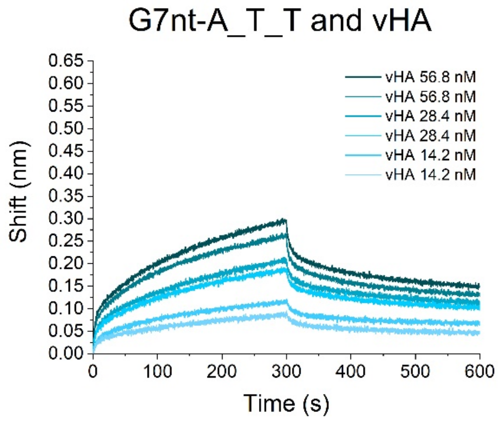

(**e**)

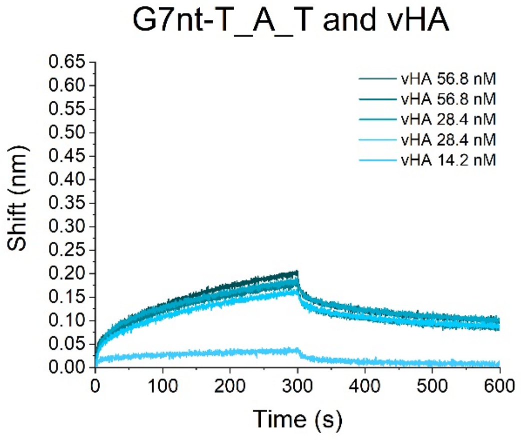

(**f**)

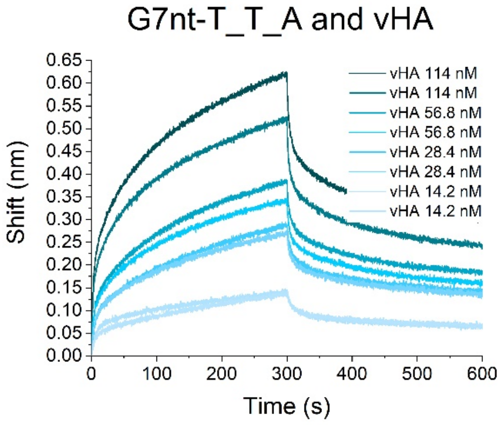
G7nt-T_T_A and vHA
Shift (nm)
Time (s)
vHA 114 nM
vHA 114 nM
vHA 56.8 nM
vHA 56.8 nM
vHA 28.4 nM
vHA 28.4 nM
vHA 14.2 nM
vHA 14.2 nM

(**g**)

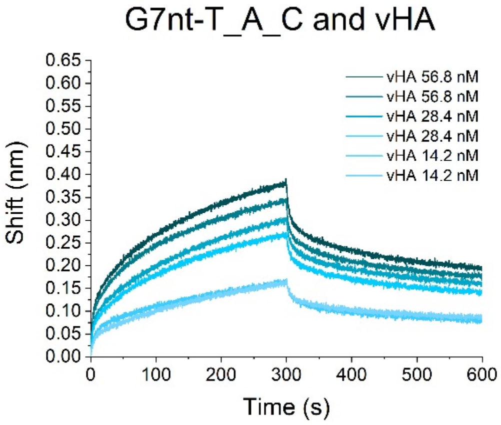
G7nt-T_A_C and vHA
Shift (nm)
Time (s)
vHA 56.8 nM
vHA 56.8 nM
vHA 28.4 nM
vHA 28.4 nM
vHA 14.2 nM
vHA 14.2 nM

(**h**)

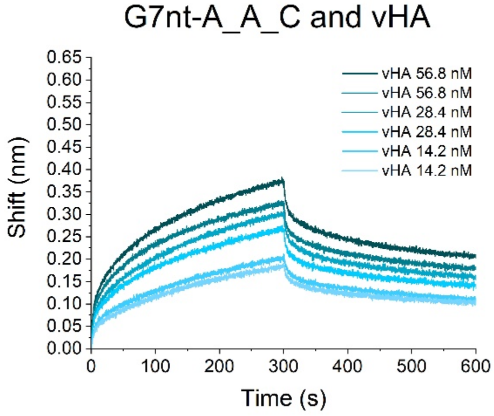
G7nt-A_A_C and vHA
Shift (nm)
Time (s)
vHA 56.8 nM
vHA 56.8 nM
vHA 28.4 nM
vHA 28.4 nM
vHA 14.2 nM
vHA 14.2 nM

(**i**)

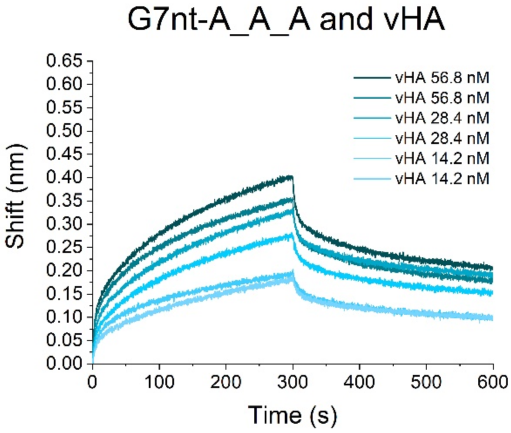
G7nt-A_A_A and vHA
Shift (nm)
Time (s)
vHA 56.8 nM
vHA 56.8 nM
vHA 28.4 nM
vHA 28.4 nM
vHA 14.2 nM
vHA 14.2 nM

(**j**)

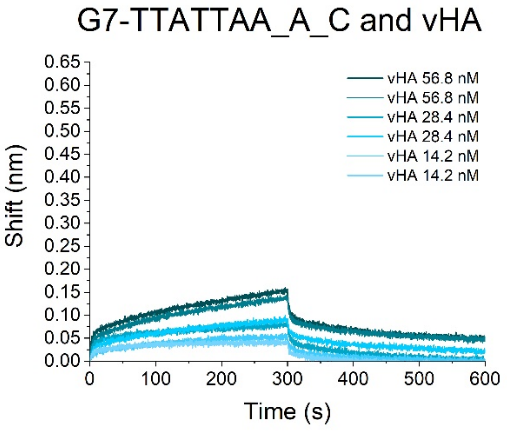
G7-TTATTAA_A_C and vHA
Shift (nm)
Time (s)
vHA 56.8 nM
vHA 56.8 nM
vHA 28.4 nM
vHA 28.4 nM
vHA 14.2 nM
vHA 14.2 nM

(**k**)

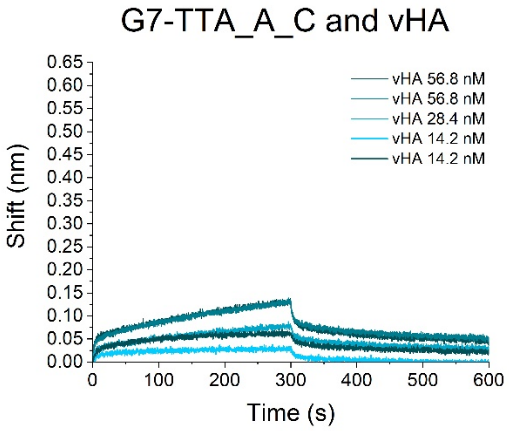
G7-TTA_A_C and vHA
Shift (nm)
Time (s)
vHA 56.8 nM
vHA 56.8 nM
vHA 28.4 nM
vHA 14.2 nM
vHA 14.2 nM

(**l**)

Supplementary Figure S2. BLI sensorgrams for complexes of aptamers with recombinant hemagglutinin rHA of A/Vietnam/1203/2004 (H5N1) strain.

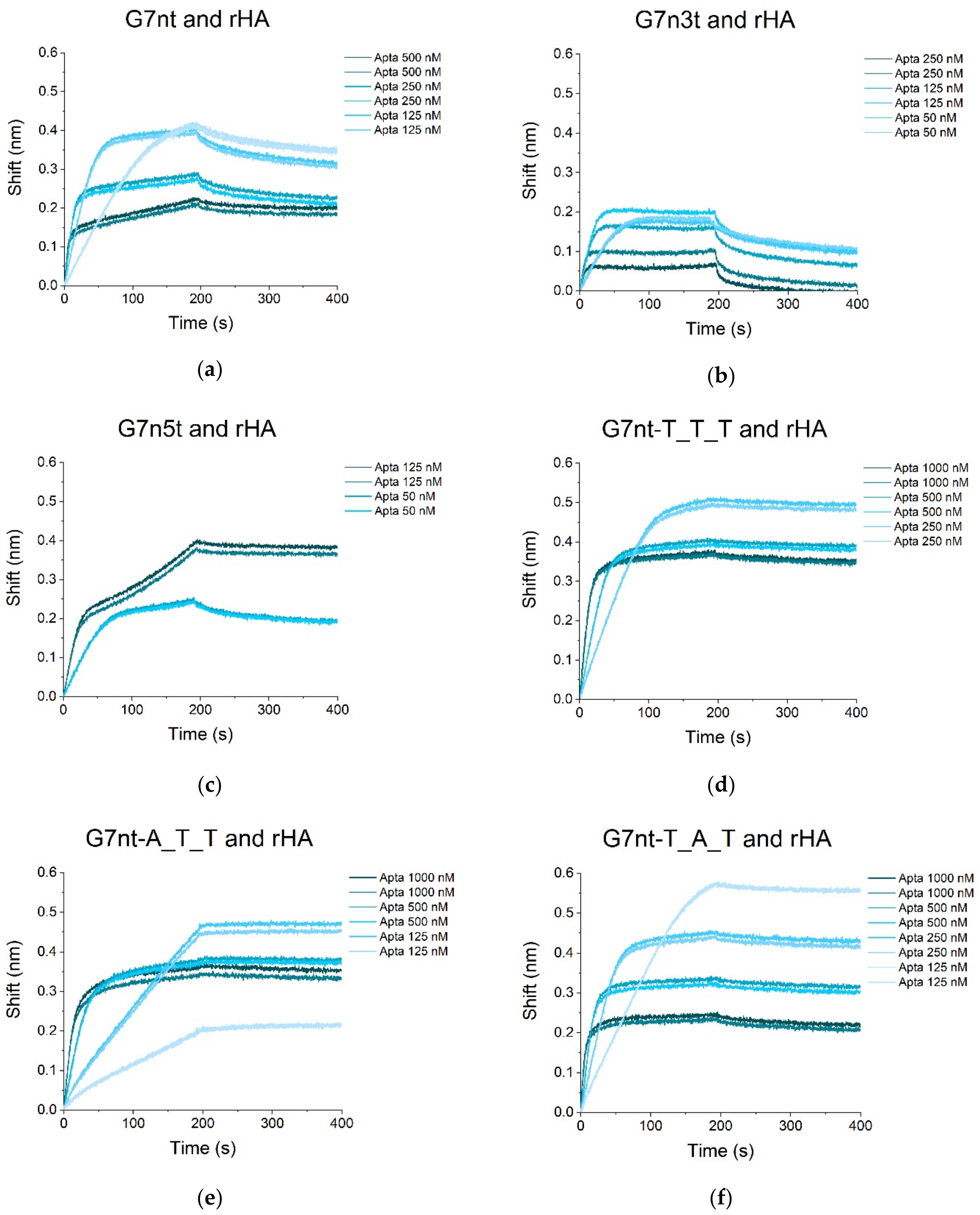

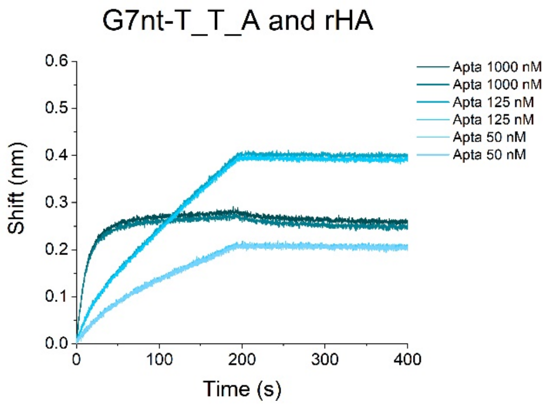

(**g**)

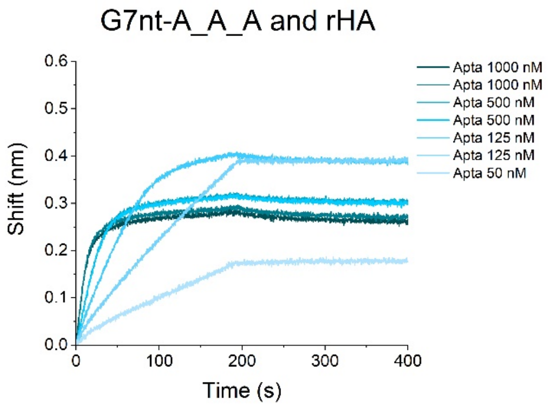

(**h**)

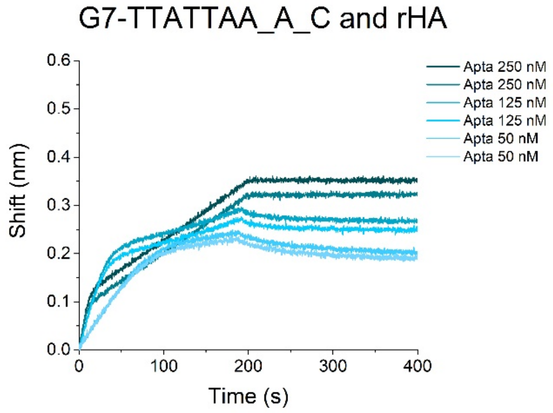

(**i**)

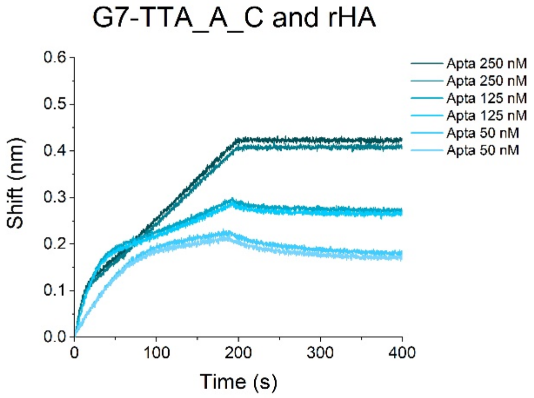

(**j**)

Supplementary Figure S3. Melting and annealing curves for G7n3t (**a**) and G7nt-A-T-T (**b**). Data for melting process shown by black squares, and annealing curve is shown by red circles.

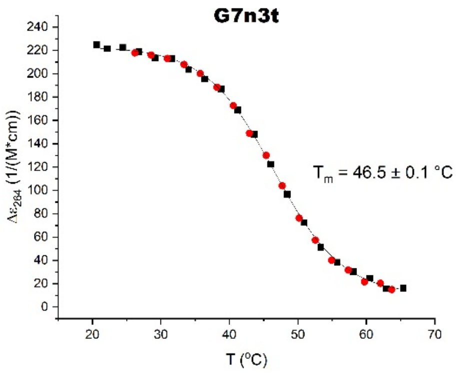

(**a**)

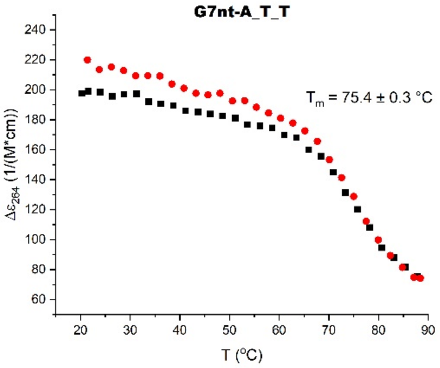

(**b**)

Supplementary Figure S4. Chromatograms of calibration oligonucleotides and calibration curve.

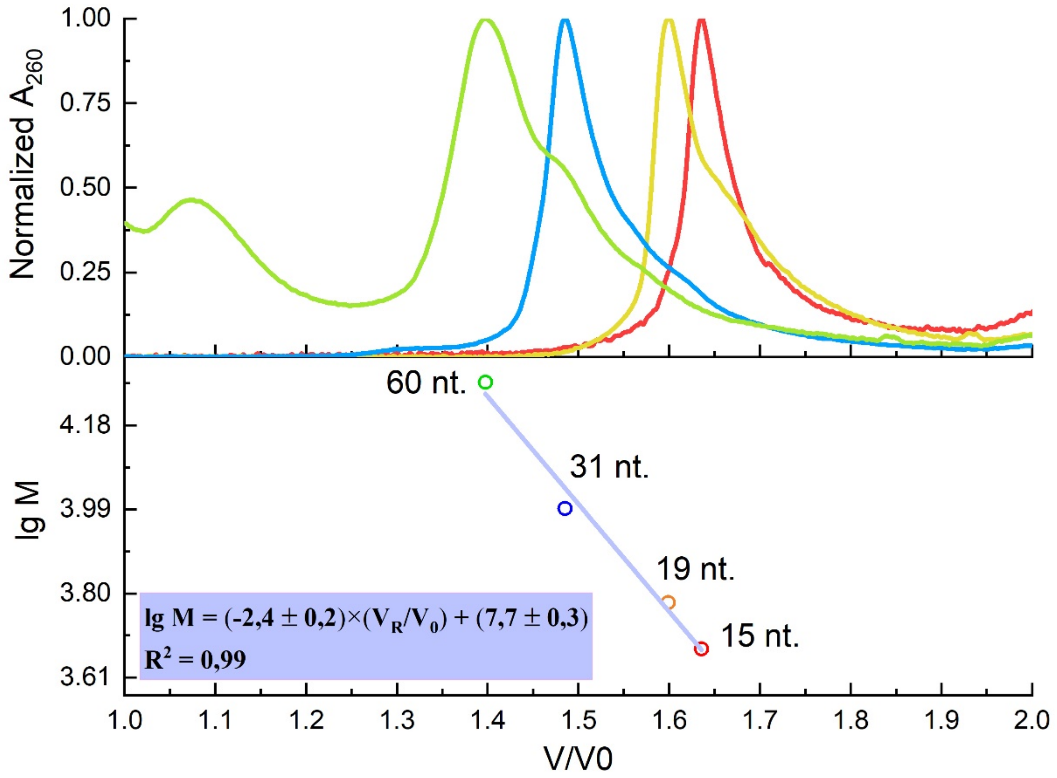

Supplementary Figure S5. Normalized chromatograms of RHA0385 aptamer and its variants. For the main peaks the values of M_{exp}/M ratio are given.

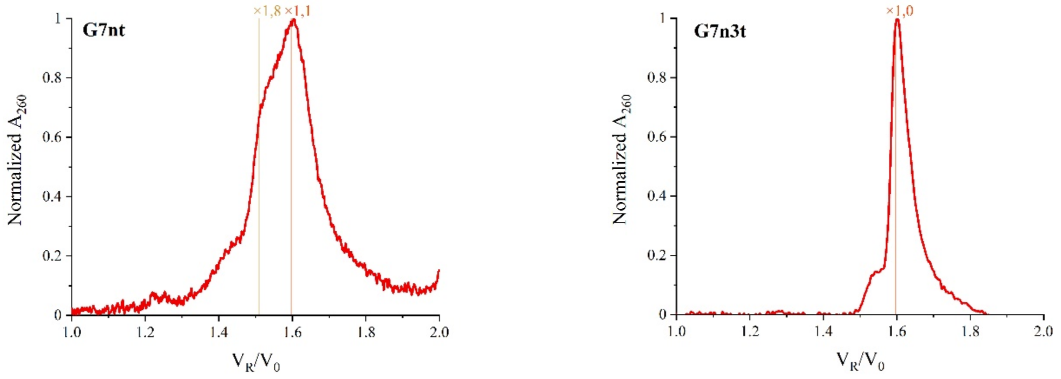

(**a**) (**b**)

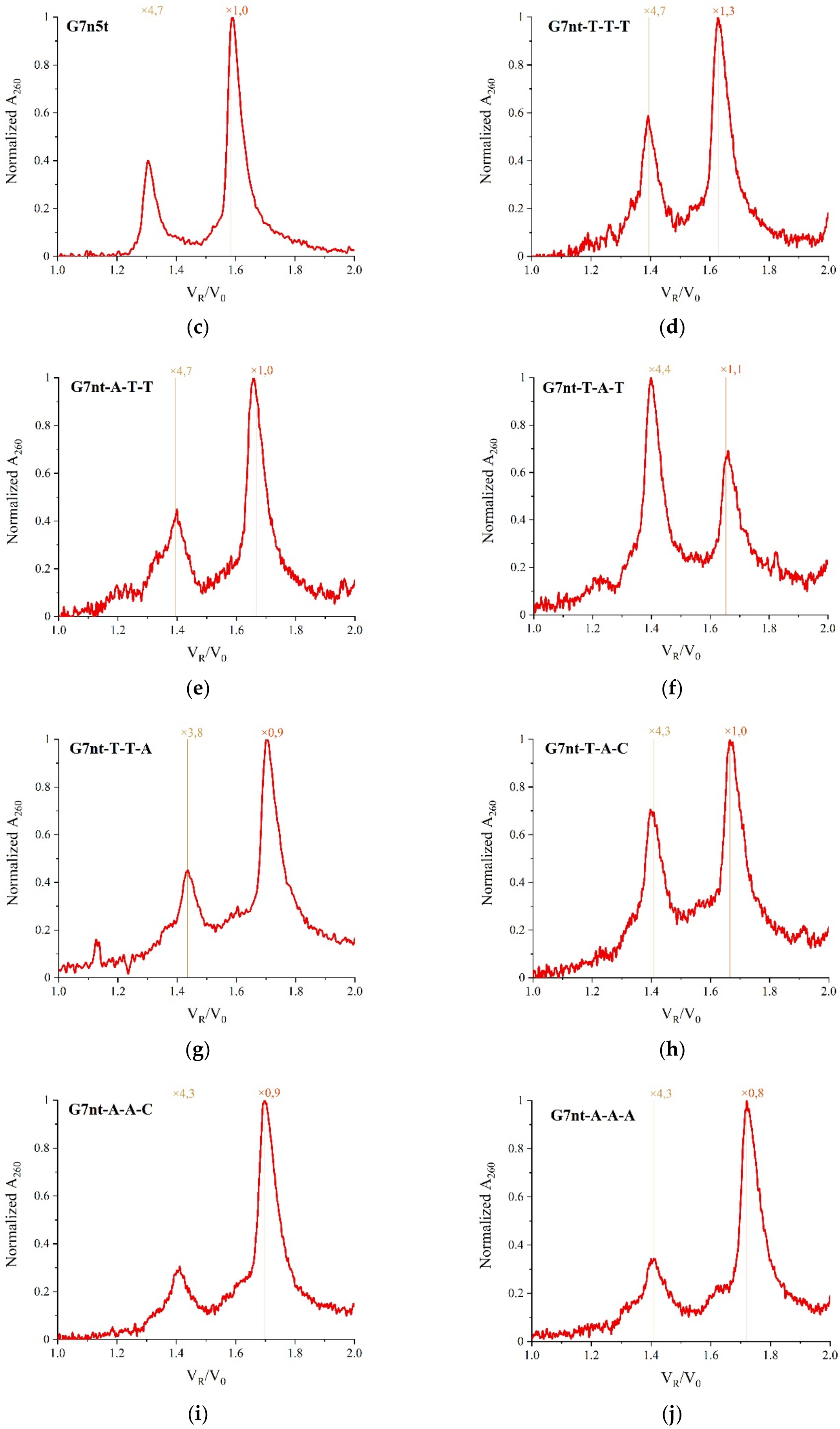

G7n5t
×4,7
×1,0
Normalized A260
VR/V0
(c)
G7nt-T-T-T
×4,7
×1,3
Normalized A260
VR/V0
(d)
G7nt-A-T-T
×4,7
×1,0
Normalized A260
VR/V0
(e)
G7nt-T-A-T
×4,4
×1,1
Normalized A260
VR/V0
(f)
G7nt-T-T-A
×3,8
×0,9
Normalized A260
VR/V0
(g)
G7nt-T-A-C
×4,3
×1,0
Normalized A260
VR/V0
(h)
G7nt-A-A-C
×4,3
×0,9
Normalized A260
VR/V0
(i)
G7nt-A-A-A
×4,3
×0,8
Normalized A260
VR/V0
(j)

Supplementary Figure S6. Plots with linearization of parameter k_{on}×C+k_{off} vs C for (a) RHA0385 –rHA and (b) RHA0385 –vHA complexes. The k_{on}×C+k_{off} is a parameter under exponential function which describes the association phase; and C is a concentration of complex partner in solution (aptamer in the case of complex RHA0385–rHA and vHA in the case of complex RHA0385–vHA).

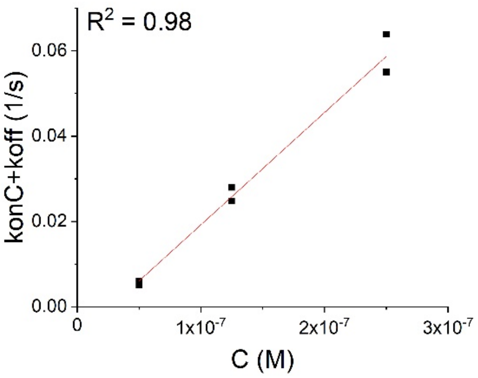

(a)

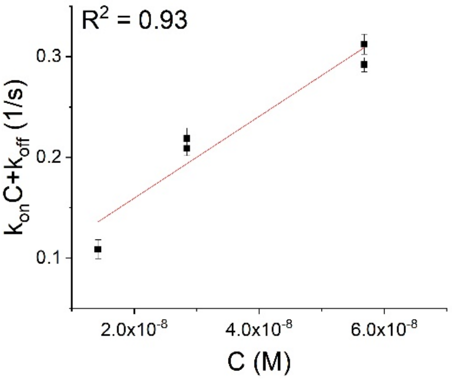

(b)
